# Application of Edible Alginate Films with Pineapple Peel Active Compounds on Beef Meat Preservation

**DOI:** 10.3390/antiox9080667

**Published:** 2020-07-26

**Authors:** Sofia C. Lourenço, Maria João Fraqueza, Maria Helena Fernandes, Margarida Moldão-Martins, Vítor D. Alves

**Affiliations:** 1 LEAF, Linking, Landscape, Environment, Agriculture and Food, Instituto Superior de Agronomia, Universidade de Lisboa, Tapada da Ajuda, 1349-017 Lisboa, Portugal; sofiaclourenco@isa.ulisboa.pt (S.C.L.); mmoldao@isa.ulisboa.pt (M.M.-M.); 2 CIISA—Centre for Interdisciplinary Research in Animal Health, Faculdade de Medicina Veterinária, Universidade de Lisboa, Avenida da Universidade Técnica, Pólo Universitário do Alto da Ajuda, 1300-477 Lisboa, Portugal; helenafernandes@fmv.ulisboa.pt

**Keywords:** antioxidant activity, active alginate films, pineapple peel, meat, lipid oxidation, microencapsulation

## Abstract

Alginate-based edible films containing natural antioxidants from pineapple peel were applied in the microbial spoilage control, color preservation, and barrier to lipid oxidation of beef steaks under storage at 4 °C for five days. Different stabilization methods of pineapple peel compounds were used before incorporation into alginate films, including extracted compounds with an hydroalcoholic solvent encapsulated in microparticles, microparticles produced by spray-drying pineapple peel juice, and particles obtained by milling freeze dried pineapple peel. Bioactive films exhibited higher antioxidant activity (between 0.15 µmol to 0.35 µmol FeSO_4_.7H_2_O/g dried film) than the alginate film without these compounds (0.02 µmol FeSO_4_.7H_2_O/g dried film). Results showed that control films without active compounds had no significant effect on decreasing the microbial load of aerobic mesophilic and *Pseudomonas* spp., while the films containing encapsulated hydroalcoholic extract showed a significant inhibitory effect on microbial growth of meat at two days of storage. Alginate films containing peel encapsulated extract were effective for maintaining the color hue and intensity of red beef meat samples. Pineapple peel antioxidants have the potential to retard lipid oxidation in meat samples, and the possibility of incorporation of a higher amount of pineapple peel bioactive compounds in the films should be investigated.

## 1. Introduction

The combination of packaging and synthetic antioxidants has been one of the main strategies for extending the shelf life of meat products after their production. However, the use of non-biodegradable and non-renewable packaging materials contributes to serious environmental problems. The application of synthetic antioxidants in food products is also a concern due to their potential toxicity and negative effects on human health. In addition, there is an increasing consumer consciousness for cleaner labels without chemical synthetic components [1,2,3,4,5,6]. To overcome these constraints, much effort has been dedicated to the development of new packaging systems resulting from the combination from edible biopolymers and natural antioxidants, such as active edible films.

Edible films are defined as a thin layer of edible material, which may be applied as a wrapping or between food components and can be consumed as a part of the product. Therefore, the materials used in the films’ formulation should be food grade and not negatively affect the organoleptic properties of the food products [7]. The most commonly used materials are from renewable sources, like proteins (e.g., gelatin, whey protein) [8,9]; polysaccharides (e.g., chitosan, starch, pectin, carrageenan, and alginate) [10,11]; and lipids (oils and waxes) [12,13]. Concerning alginate, it is extracted from brown algae, mainly *Laminaria hyperborean*, *Macrocystis pyrifera*, *Ascophyllum nodosum*, and, to a lesser extent, from *Laminaria digitate*, *Laminaria japonica*, *Eclonia maxima*, *Lesonia negrescens*, and *Sargassum* sp. [14]. It is a water-soluble polysaccharide composed of β-D-mannuronic acid (M) and β-L-guluronic acid (G) linked by 1-4 glycosidic bonds. These monomers may be arranged along the polymer chain in three types of block structures (M, G, and MG blocks). The chemical structure, G/M ratio, and average molecular weight are dependent on the type of algae and harvesting season [15,16]. It has been widely used in the food industry as a thickening, stabilizing, suspending, gel forming, and emulsion stabilizing agent [16,17]. In addition to that, due to its film-forming ability, alginate has been widely studied as material for the development of edible and biodegradable films for food packaging [17,18,19].

A wide range of natural compounds, such as antioxidants and antimicrobials, can be incorporated into packaging materials to improve their functionality, turning them into active barriers. This strategy has been studied by combining edible biopolymers with compounds from aqueous plant extracts, essential oils, and those naturally present in particles produced by milling dried agro-industrial residues [11,20,21]. Concerning the use of alginate as biopolymer with natural compounds, Mahcene et al. [22] developed active films with the incorporation of essential oils from several medicinal plants, which have shown considerable antioxidant and antibacterial activities. In addition, Fabra et al. [10] reported the production of active alginate-based edible films containing phenolic extracts from green tea and grape seed that exhibited antioxidant and antiviral activities. Rezaei et al. [23] demonstrated the effect of apple peel extract in alginate films in the inhibition of microbial growth and increasing the shelf life of sauced silver carp fillets during refrigerated storage.

The application of active edible films in the preservation of meat products has been extensively reviewed [24,25]. As examples of recent works, Farhan et al. [26] applied active edible packaging films based on semi-refined κ-carrageenan incorporated with a water extract of germinated fenugreek seeds, with antimicrobial and antioxidant activities, to extend shelf-life of fresh chicken breast. Esmaeili et al. [27] developed chitosan and whey protein films combined with free or nanoencapsulated garlic essential oil to extend the shelf life of refrigerated vacuum-packed sausages. These active films retarded lipid oxidation and the growth of the main spoilage bacterial groups studied.

Concerning the sources of natural active molecules, attention has also been focused on byproducts and residues from agro-industrial processes, which is an economical and practical way of obtaining potent antioxidants and antimicrobials along with the valorization of those residues still rich in functional compounds that are usually discarded. In this context, a previous work focused on the valorization of pineapple peel by producing an extract sowing a stable antioxidant activity over six months after microencapsulation by spray drying [28]. Considering the advantages of incorporating natural antioxidants for extending the applications of edible films, the main objectives of the present work were to develop for the first time bioactive alginate films enriched with pineapple peel bioactive compounds and to study their application in the preservation of meat. In most of the works presented in the literature, the active compounds are first extracted from the plant materials before being incorporated in the films. However, the extraction step may not recover important compounds that may remain in the plant matrix. In addition, due to their high reactivity, the compounds present in the extract may be degraded once removed from the protective plant matrices. As such, in this work, pineapple peel bioactive compounds were added to the films after being extracted and encapsulated in microparticles, loaded in microparticles produced by spray-drying pineapple peel juice, and present in particles obtained by milling freeze-dried pineapple peel. The effect of different bioactive compounds stabilization methods was studied to determine the potential of these active films to retard the color change and lipid oxidation of meat during storage under refrigerated conditions.

## 2. Materials and Methods

### 2.1. Materials

Most of the reagents and reference standards were from Sigma-Aldrich (St. Louis, MO, USA) or VWR International (Darmstadt, Germany). The peptone water and tryptone glucose extract agar were obtained from Scharlau (Barcelona, Spain). The pseudomonas agar base and the supplement cetrimide-fucidin-cephaloridine were purchased from Oxoid (Basingstoke, UK). Food-grade sodium alginate was acquired from Quimidroga, s.a, Lisboa, Portugal with a 200 mesh particle size. The solvents used, ethanol and methanol, were purchased from Panreac (Barcelona, Spain). The 2,4,6-Tris(2-pyridyl)-s-triazine (TPZ) was purchased from Alfa Aesar (Tewksbury, MA, USA).

### 2.2. Raw Materials

Fresh pineapple peel (Sweet Gold variety) was provided by Campotec IN, Torres Vedras, Portugal. This byproduct was transported under refrigerated conditions to the laboratory and was separated in small portions and stored in bags under vacuum at −80 °C until further analysis. Three independent beef chuck eye cut samples from different young bulls, packaged under vacuum, were purchased from local meat logistic distribution (Santarém, Portugal).

### 2.3. Pineapple Peel Extract Preparation and Microencapsulation

Pineapple peel extract was prepared as described by Lourenço et al. [28]. Briefly, a solid–liquid extraction process was applied with minced pineapple peel and a water:ethanol mixture (20:80 *w*/*w*) as solvent. The supernatant was separated, and the ethanol was evaporated using a rotatory evaporator (Rotavapor R II—Buchi, Flawil, Switzerland). The final aqueous solution (extract) was stored away from light. The pineapple peel extract was stabilized by microencapsulation using the spray drying process, as described elsewhere [28]. The feed solution, prepared by adding maltodextrin (wall material) to the aqueous extract (5% *w*/*w*), was dried at an inlet air temperature of 150 °C. The dried particles were collected and stored in the dark at 5 °C.

### 2.4. Production of Peel Juice Microparticles and Peel Freeze-Dried Powder

Pineapple peel was feed into a cold press juicer machine. The liquid recovered from the peel was centrifuged at 1000 rpm for 15 min at 15 °C (HERMLE Labortechnik 383 K, Gosheim, Germany). Afterwards, maltodextrin was added to the supernatant. The amount added was chosen in order to obtain a ratio mass of maltodextrin/phenolics content in the juice equal to that of the feed used in the previous section, and the mixture was spray dried using the same process conditions. Dried particles were collected and stored in sealed amber flasks in the dark at 5 °C. The freeze-dried powder was produced by freezing pineapple peel in an ultra-freezer at −80 °C for 48 h that was freeze-dried (Scanvac CoolSafe, Lillerød, Denmark) for seven days. The freeze-dried material was ground in a mill (Pulverisette 14 Premium, Fritsch, Idar Oberstein, Germany) with a 0.710 mm sieve. The powder was collected and stored in sealed amber flasks in the dark at 5 °C.

### 2.5. Production of Alginate Films

Alginate solutions with a concentration of 1% (*w*/*v*) were prepared by dissolving the alginate powder in distilled water under constant stirring at room temperature. After complete dissolution, glycerol was added as a plasticizer (50% *w*/*w*, alginate basis), and the mixture was stirred with an Ultra Turrax homogenizer (IKA Turrax Digital, Model T25 basics, Staufen, Germany) for 3 min at 1500 rpm to obtain a homogenous solution. Depending on the type of film to be prepared, the different microparticles and the powder were added to the filmogenic solutions (30% *w*/*w*, alginate basis) under magnetic stirring for 10 min. The resulting solutions were placed in an ultrasounds bath for 30 min to remove the entrapped air bubbles. The film-forming solutions were cast in Petri plates and dried at 40 °C for 12 h under ventilation (Binder, Model D, Baddeckenstedt, Germany). After drying, the films were peeled off from the casting surface, and the obtained stand-alone films were sprayed with a crosslinking solution (calcium chloride 6% *w*/*v*) in order to add 3.2 mg Ca^2+^/cm^2^ of dry film. After that, the films were left to dry at 40 °C for 5 min and were conditioned in desiccators with silica gel before analysis. The concentration of alginate, glycerol, and CaCl_2_ were chosen based on preliminary studies where suitable mechanical properties of the films were guaranteed (results not shown).

Four different films were prepared—alginate with microparticles with encapsulated hydroalcoholic extract (EF), alginate with pineapple peel juice microparticles (PJF), alginate with freeze-dried peel powder (FDF), and control films only with alginate (AF).

### 2.6. Total Phenolic Content and Antioxidant Properties

#### 2.6.1. Extracts of Powder and Microparticles

Extracts from powder and microparticles were obtained by the method described by Rocha et al. [29] with some modifications. The spray dried particles and powder (200 mg) were dispersed in 10 mL of methanol and strongly homogenized with an Ultraturrax homogenizer (IKA Labortechnik T25 Basic, Staufen, Germany) at 13,500 rpm for 3 min to break the particles. The suspension was left at 10 °C in the dark for 12 h. Then, the solutions were centrifuged at 7000 rpm for 15 min at 4 °C, and the collected supernatant was stored in amber glass flasks until measuring the total phenolic content and the antioxidant activity.

#### 2.6.2. Total Phenolic Content and Ferric Reducing Antioxidant Power

Total phenolic content (TPC) of microparticles and powder was determined by direct measurement of the absorbance of the respective extracts at 280 nm as described by Lourenço et al. [28]. The particles and powder loading were expressed as mass of gallic acid equivalents (GAEs) per mass of dry solids (mg GAE/g of dry solids). The ferric reducing antioxidant power (FRAP) method was performed according to Benzie et al. [30] with some modifications. Briefly, a 90 µL aliquot of the extracts obtained as described in the previous section or 3 cm^2^ of each film was transferred to glass tubes, added up with 270 µL of deionized water, and reacted with 2.7 mL of the working FRAP solution. After reaction, absorbance was measured at 595 nm (UNICAM, UV/Vis Spectrometer—UV4, Alva, UK). FeSO_4_.7H_2_O (500–2000 µM) was used as reference. The antioxidant activity was expressed as µmol FeSO_4_.7H_2_O/g dry sample.

### 2.7. Evaluation of the Antioxidant Effect of Alginate Films on Meat During Storage

**Figure 1:**
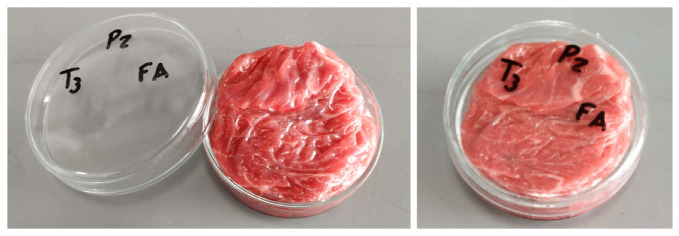
Meat wrapped with alginate films in the Petri dish.

From commercial beef chuck eye, a mass of approximately 20 g of meat was cut, wrapped with a film sample, and introduced on a glass Petri dish that was closed with its lid (Figure 1). For each day of analysis, a triplicate was prepared for each type of film. The samples were stored at a constant temperature of 4 °C in a refrigerated chamber without light. Analyses were performed at four time points—on day 0 and after two, three and five days of storage. Control samples were also stored at the above conditions without the presence of any film.

To evaluate the protective effect of each film over time, the meat of each glass dish was aseptically unwrapped and cut in the middle. One part (half of the same sample) was selected for the determination of counts of total aerobic mesophilic bacteria and *Pseudomonas*, and the other half for assessment of the meat color and lipid oxidation.

### 2.8. Microbial Analysis

For the microbial counts, the preparation of samples, initial suspension, and decimal dilutions were done according to International Standards Organization (ISO 6887-2:2017) [31]. Briefly, 10 *g* of meat were transferred into sterilized stomacher bags, diluted with 90 mL of peptone water, and stomached for 90 s in a Stomacher Masticator (Bagmixer 400P Interscience, St Nom la Bretèche, France), resulting in a 10^−1^ dilution in agreement. Decimal dilution series were prepared in peptone solution, and 1 mL from each dilution was plated on the surface of the Petri dishes. Total mesophiles counts were performed in tryptone glucose extract agar, incubating at 30 °C for 48 h [32]. For the pseudomonas, aliquots of 0.1 mL of serial dilutions were spread onto Pseudomonas Agar Base, with supplements cetrimide-fucidin-cephaloridine and were incubated at 30 °C for 48 h [33]. The results were expressed in log_10_CFU.g^−1^ meat.

### 2.9. Color Measurement

At each storage time, meat surface color measurements were performed with a Minolta CR-300 colorimeter (Minolta CR-300, Chromometer, Osaka, Japan) using the coordinates L*, a*, and b* of the CIELAB color system (CIE,1976). The chroma (C*) was obtained by (a*^2^ + b*^2^)^1/2^ and Hue angle by h° = arctan (b*/a*) × 180/π. The instrument was calibrated against a white tile ceramic reference according to manufacturer instructions. For each sample, two different analyzes were carried out. First, the color was measured before opening the Petri dishes, simulating the use of transparent food packaging. The second analysis was done directly on the meat surface 10 min after opening the dishes and films removal. Three random readings at different locations per sample were taken and averaged.

### 2.10. Lipid Oxidation

Secondary products of lipid oxidation in the meat samples were evaluated using the 2-thiobarbituric acid (TBARS) test method proposed in the literature [34,35,36]. In this work, 15 *g* of meat sample was dispersed in 7.5% trichloroacetic acid (30 mL) and homogenized in an UltraTurrax (IKA T25 basic, Staufen, Germany) for 2 min. The homogenate was then filtered through a filter paper Whatman No. 1. The filtrate was reacted with 0.02 M TBA and incubated in a boiling water bath for 40 min. The absorbance was measured at 530 nm. The TBARS index was calculated from a standard curve of malonaldehyde (MDA) with 1,1-3,3 tetraetoxipropane and expressed as mg MDA/kg of meat. Duplicates of the same sample were performed.

### 2.11. Statistical Analysis

The design of the experiment considered three cut batches × five package conditions (control + four edible films formulations) × days of storage. The evaluation of storage time was considered at four time points: on the first day (day 0), and after two, three, and five days of storage. The results were evaluated by analysis of variance (ANOVA) considering as main factors the different conditions of film production and Tukey’s test with a 0.05 significance level using StatisticaTM v.8.0 Software (StatSoft Inc., 2007, Tulsa, Oklahoma, US).

## 3. Results and Discussion

### 3.1. Total Phenolic Content and Antioxidant Properties

The highest value of TPC was observed for freeze-dried powder (7.48 ± 0.05 mg GAE/g dry solids) followed by the microparticles with encapsulated hydroalcoholic extract (4.95 ± 0.01 mg GAE/g dry particles) and the peel juice (3.77 ± 0.02 mg GAE/g dry particles). By the FRAP method, the antioxidant of the freeze-dried powder (116.43 ± 1.85 µmol FeSO_4_.7H_2_O/g dry powder) is statistically higher than the value of microparticles with encapsulated extract (79.25 ± 2.36 µmol FeSO_4_.7H_2_O/mg dry particles) and peel juice microparticles (72.94 ± 1.47 µmol FeSO_4_.7H_2_O/mg dry particles). It can be observed that the incorporation of both (microparticles and powder) into alginate films increased significantly (*p* < 0.05) the antioxidant activity compared to that of the control (only with alginate) (Table 1).

Alginate films with freeze dried peel powder had a significantly higher antioxidant activity than those with both types of microparticles. This fact is attributed to its higher TPC content, as the same mass of microparticles or powder was added in all film formulations (30% *w*/*w* alginate basis). A larger percentage of microparticles should be added compared to the powder addition. Generally, the antioxidant capacity is proportional to the amount of sample added to the films [10,37,38]. Thus, for an increase in antioxidant activity, a higher amount of powder and microparticles should be tested. Nevertheless, these novel films present a good potential to be applied as active barriers with antioxidant activity.

### 3.2. Antimicrobial Activity of Films

**Figure 2:**
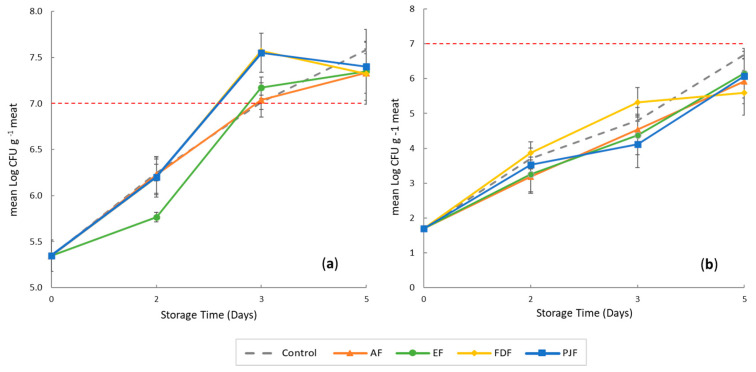
Effect on microbial load of alginate film and alginate film with microparticles and pineapple peel powder during five days of storage of meat at 4 °C. (**a**) Total aerobic mesophilic bacteria and (**b**) *Pseudomonas* spp. Alginate film with microparticles with encapsulated hydroalcoholic extract (EF), alginate film with pineapple-peel-juice microparticles (PJF), alginate film with freeze-dried peel powder (FDF), and control films only with alginate (AF).

The results of total mesophiles counts on meat samples wrapped with alginate film and alginate film with microparticles and pineapple peel powder during five days of storage are shown in Figure 2.

The initial microbial load of mesophilic in all beef cuts was approximately 5.35 log CFU/g (Figure 2a). The mesophilic counts include the microorganisms responsible for spoilage of meat, and it will also give an indication of the of the meat quality maintenance. This initial count in meat samples is due to the fact that the cut was packaged under vacuum and stored in refrigeration. The main groups involved in this count should be acid lactic bacteria (Gram-positive) more than Gram-negative. According to Mansur et al. [39], bacterial species belonging to *Pseudomonadaceae* (*Pseudomonas* spp.) and lactic acid bacteria (*Lactobacillus* sp.) dominated the bacterial communities in beef stored under air and vacuum package. In fact, *Lactobacillus* sp. in vacuum-package-stored beef samples increased along with storage time, reaching > 75% of the total population on days 7–21 of storage [40,41].

The meat cuts samples used in this experiment followed the normal commercial operations in which the beef arrives under vacuum from slaughtering industries where beef carcasses were slaughtered, deboned, cut, packaged, and driven to retail distribution. Then, the cuts are removed from the package and sliced being repackaged again with a modified atmosphere package. So, these initial mesophilic counts are in the range of the expected results (4–5 log CFU/g).

In the present study, the meat after the vacuum package removing was stored wrapped in films without any modified atmosphere at a refrigeration temperature of 4 °C.

Mesophilic counts in all meat film groups increased significantly during storage time. Nevertheless, at day 2 of storage, the results showed that meat samples wrapped with films containing hydroalcoholic extract (EF) present a significantly (*p* < 0.05) lower microbial counts when compared to the control and the other films. The incorporation of ethanolic pineapple-peel extract encapsulated in microparticles into films showed to be more effective than the other pineapple-peel bioactives stabilization methods to inhibit the increment of total mesophilic values after two days of storage. This fact may be attributed to a more adequate release rate of encapsulated phenolic compounds from the microparticles when compared to their release from peel freeze-dried powder and peel juice particles, enabling a higher antimicrobial activity of these films for these microorganisms [42,43,44].

Also, after three days of storage, a significantly lower (*p* < 0.05) microbial growth of mesophilic groups in samples with AF films, similar to that of control samples without films, was perceived. In addition, films with encapsulated hydroalcoholic extract also present a superior antimicrobial activity against mesophilic groups when compared to films with freeze-dried powder and peel juice particles. This fact may be attributed to the chemical composition of the bioactive particles and powder. Due to their production methodology, beyond the peel antimicrobial compounds, freeze-dried powder and peel juice particles are expected to contain other peel low-molecular-weight molecules, such as reducing sugars, which may have been released to meat surface and used as extra carbon source resulting in a slightly higher growth of mesophilic groups after three days. Peel reducing sugars are less likely to be present in films with encapsulated hydroalcoholic extract due to their low solubility in ethanol.

Nevertheless, after three days of refrigerated storage, the total mesophilic bacteria reached the value of 7 log CFU/g in all samples, which is above the acceptable limit [40,41,45,46]. The results of the present study showed that the use of these films could not successfully reduce the growth of mesophilic bacteria until the end of the study period. The final bacterial counts levels in meat samples were found 7.5 log CFU/g indicating that after five days of refrigerated storage at 4 °C, this meat was not in the microbial acceptable range due to the occurrence of different biological activities.

*Pseudomonas* is a Gram-negative bacterium comprising the most common spoilage microorganisms in meat stored in refrigeration temperatures under aerobic conditions [47,48]. As shown in Figure 2b, the initial count of *Pseudomonas* spp. was 1.69 log CFU/g, which increased during storage and reached a final population of 6.68 log CFU/g for control samples, whereas for films was reached a value near 6.2 log CFU/g. These counts showed that all the samples never reached the 7 log CFU/g value throughout the storage period independently of the different study conditions. The behavior of *Pseudomonas* spp. count was different from mesophilic counts. The addition of films to the meat does not significantly (*p* > 0.05) delay the growth of *Pseudomonas* spp. compared to control samples (without any film) in the different days of storage.

There are no reports of studies that have evaluated the application of pineapple peel extracts in meat or active films. According to the literature, few studies of natural aqueous extracts from fruit sources in films are reported [25]. Adding active compounds to films such as herbs, spices, or essential oils showed to be more effective in delaying microbial proliferation and growth in meat products [49,50]. However, films containing spices and essential oils may impart negative sensory attributes that are not accepted by consumers. This fact is generally minimized when using plant materials that do not present flavors as strong as those used in this work. Also, when the meat was packaged without any modified atmosphere, all the microbial groups showed viable counts higher than those of referred monitoring packaging conditions [47]. In this study, the meat arrived cut and vacuum-packed, according to the current conditions of commercialization of these pieces, presenting high counts of lactic acid bacteria.

Although the effect of edible films may be due to their phenolic content, some of these compounds can impart antioxidant activity without presenting antimicrobial properties. The results obtained reflect that pineapple peel in powder forms did not lead to an antimicrobial effect of aerobic mesophilic and *Pseudomonas* spp. on meat samples, although films containing encapsulated hydroalcoholic extract showed a significant inhibitory effect on total aerobic mesophilic bacteria growth until two days of storage. In addition, there is the possibility that these edible films may improve the preservation of meat through their antioxidant activity presented in Section 3.1.

### 3.3. Color

**Figure 3:**
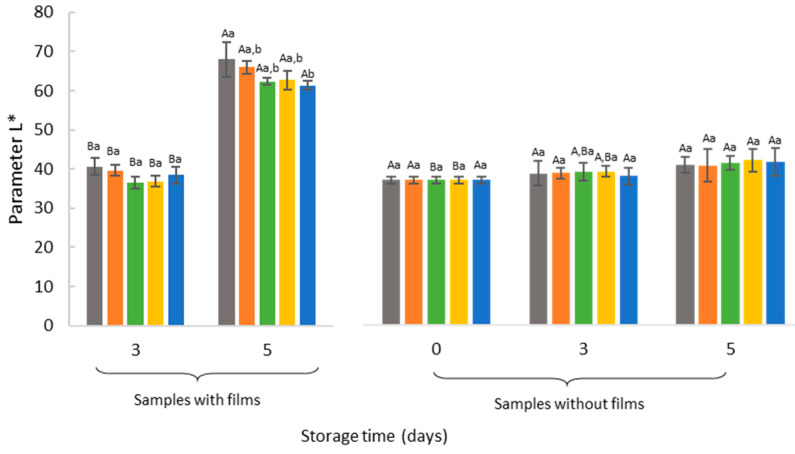

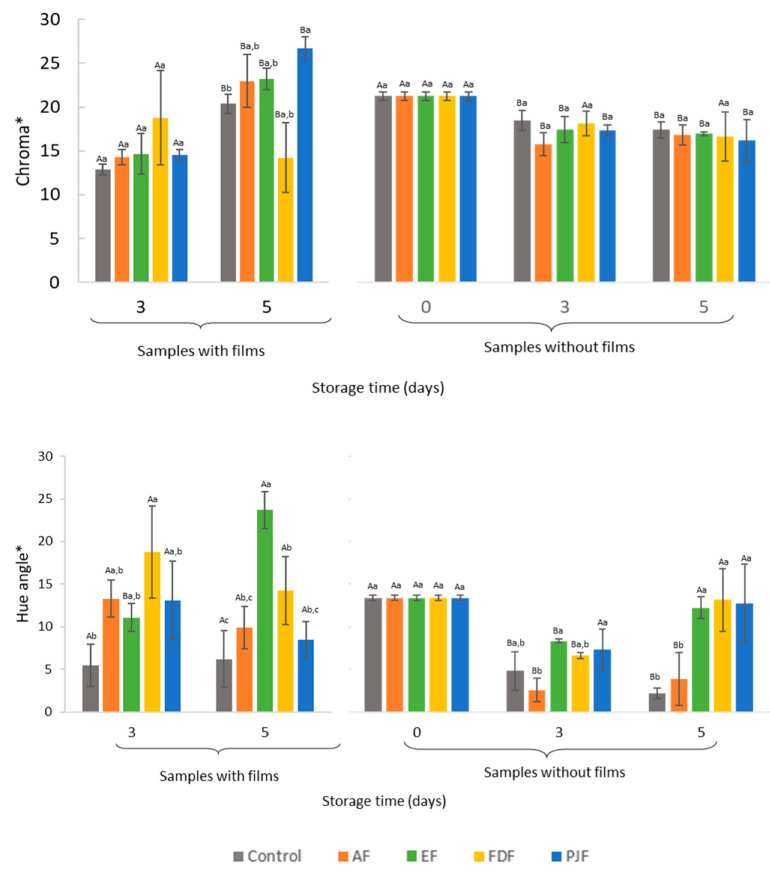
Color parameters of the samples with or without films after five days of storage at the refrigerated conditions. Different lowercase letters represent a statistically significant difference among results for the same storage time (*p* < 0.05). Different capital letters represent statistically significant parameter values of the same sample over the storage period (*p* < 0.05). Alginate film with microparticles with encapsulated hydroalcoholic extract (EF), alginate film with pineapple-peel-juice microparticles (PJF), alginate film with freeze-dried peel powder (FDF), and control films only with alginate (AF).

In the development of new films to extend meat shelf life, success cannot be achieved if the color attribute is negatively affected. Besides, consumer decisions on the purchase of fresh meat are mostly based on color. When interrupting the equilibrium between in vivo prooxidative and antioxidant system, the oxidative reactions occurs in the post-slaughter stage during meat processing and storage in which deterioration and consequently discoloration of meat is notice [51]. The typical form of myoglobin, the principal protein responsible for meat color, associated with oxygen concentration can influence meat purchasing decisions [52]. Therefore, for each storage time changes in the color of meat samples were evaluated, and the results are shown in Figure 3.

As shown in Figure 3, for all samples measured with films on the meat surface and control samples, L* significantly increased (*p* < 0.05) during storage, indicating an increase in light reflectance [53]. This suggests that the luminosity is affected by the accumulation of exudate between the meat and the glass dish, which increases the reflectance of light in all samples. According to similar results found in the literature, these increase of L* due to the exudates released can be explained by the protein denaturation and protein conformation [54]. Oxidative damage during storage causes a structural and functional alteration of the meat proteins that may result in interactions (protein-protein such as formation or polymerization of aggregates) and modification of the amino acid chains [55]. The protein denaturation or proteolysis occurring during meat storage and maturation produces a release of water from the meat that may soak in the meat surface and accumulate on glass dishes, resulting in the dispersion of more light during color measurements [56,57,58,59]. However, at day 5 of storage, a decrease in the magnitude of lightness of samples with films compared to the control was noticed. Samples that have PJF on the meat surface revealed a significantly (*p* < 0.05) decrease in L* values compared to the control sample demonstrating some film application effect. Similarly, this conclusion can be demonstrated in samples measured after films removal. In these samples, L* values remain constant over storage time in all samples when films were removed from the meat surface. Since the effect of the glass dish disappears, it was noticed that luminosity cannot be explained by the presence of the films on the meat surface. Nevertheless, in the wrapped meat with EF and FDF, there is a significant increase (*p* < 0.05) in reflectance from day 0 to day 5 of storage.

**Figure 4:**
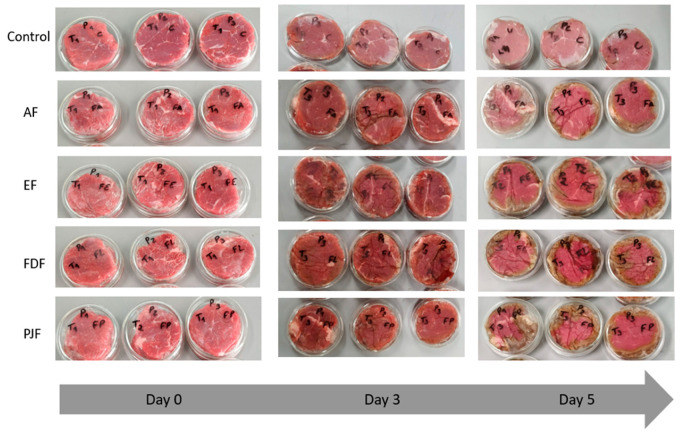
Visual appearance of color meat samples changes during the five days of storage at refrigerated temperature (4 °C). Alginate film with microparticles with encapsulated hydroalcoholic extract (EF), alginate film with pineapple peel juice microparticles (PJF), alginate film with freeze-dried peel powder (FDF), and control films only with alginate (AF).

Although L*, a*, and b* parameters could reflect important color changes, meat color is better described by its saturation proprieties (chroma) and hue angle. Chroma represents the color intensity describing how vivid is the sample color [53]. According to the results, the samples measured with the films showed a more vivid color compared to the control samples. The higher C* value of samples with films was more significant (*p* < 0.05) after five days of storage. It may be related to the gradual release of active compounds present in the films, facilitated by films swelling due to the absorption of water, due to their hydrophilic nature. When released, the active compounds can accumulate in the meat exudates. This assumption is in line with results of Figure 4, in which on the fifth day of storage, meat samples with films revealed a more intense and darker (lower L* parameter) red color than control samples. However, in Figure 4, it is possible to observe that, at day 5 of storage, a small brownish area appears on the borders of the meat due to the oxidation. These brownish border areas can occur due to the loss of film adherence in the meat borders over time.

Upon color assessment after film removal, the contact of meat samples with oxygen during 10 min before the measurement may cause the oxygenation of heme iron and the formation of vivid rose oxymyoglobin; this could depend of the myoglobin state [6,57]. After removing the films, it may be observed that the chroma of the samples decreases during storage, except for samples that were wrapped in FDF. In this case, the red intensity remains constant until the end of the storage period (Figure 3). The higher antioxidants content of freeze-dried powders may have slowed down the oxidation process.

The hue angle measurement has revealed the same conclusions (Figure 3). There is a significant decrease (*p* < 0.05) in color tone of control and AF samples after films removal and the meat exposed to oxygen, compared to the other samples. In the samples wrapped with films containing powder and microparticles, the value of hue angle measured after films removal from the meat surface at day 0 is similar to that obtained after five days of storage, meaning that films are being effective in some extent in maintaining the meat hue.

Petrou et al. [60] suggests that the presence of antioxidants such as those from oregano in chitosan films were effective to maintain and even increase the redness of chicken breast meat. In the present study, the application of films with natural antioxidants from pineapple peel extract also leads to an effective delay of meat samples oxidation and discoloration. The maintenance of hue and red intensity of the meat color over time is a sign of freshness, which could lead to a more thoroughly attractive appearance for the consumers acceptance, when films are present in food packaging. Still, the amount of powder and microparticles added to the alginate films is not enough to avoid some color changes during storage. Serrano-León et al. [49] also reported no major changes in the characteristic chicken color with the application of chitosan films containing natural antioxidants from peanut skin and pink pepper residue extract, compared to the control, at the end of storage time. These authors argued that, even though the extracts are relevant source of natural antioxidants, they should be applied in higher concentrations in future studies.

### 3.4. Inhibition of Lipid Oxidation in Meat

**Figure 5:**
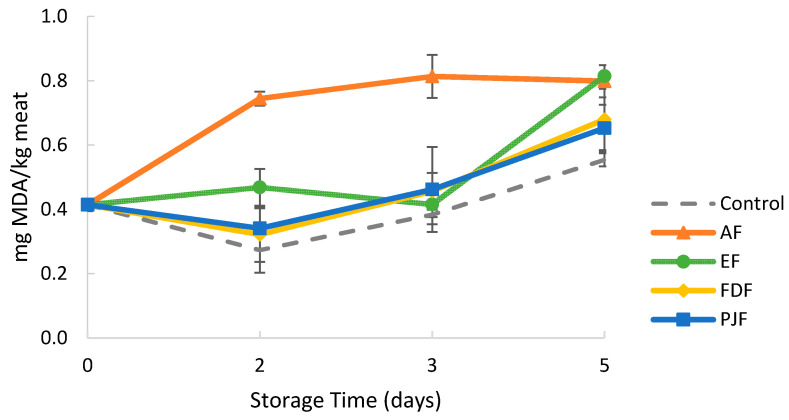
2-thiobarbituric acid (TBARS) values of meat samples during five days of storage of meat at 4 °C. The results were expressed as mean malonaldehyde (MDA) concentration in milligram per kilogram of meat. Alginate film with microparticles with encapsulated hydroalcoholic extract (EF), alginate film with pineapple-peel-juice microparticles (PJF), alginate film with freeze-dried peel powder (FDF), and control films only with alginate (AF).

Malondialdehyde (MDA) is one of the molecules produced during the oxidation of polyunsaturated fatty acids and it is considered a secondary oxidation product after lipid hydroperoxides. The ability of the films to inhibit oxidation of lipids on meat was evaluated by thiobarbituric acid reactive substance (TBARS), where higher MDA values indicate an increased oxidation. As shown in Figure 5, the TBARS values increased during storage, reaching a maximum value of 0.81 ± 0.03 mg MDA/kg meat at five days. The increase in TBARS value may be attributed to the partial exudation of meat and the increased oxidation of unsaturated fatty acids. However, all samples exhibited TBARS values bellow 2.0 mg MDA/kg, which is considered as the threshold of oxidative rancidity perception in beef meat [61].

The TBARS value of all samples and control increased sharply after the third day of storage. The exception was the TBARS value of AF samples which increased since the beginning, reaching a plateau around 0.8 mg MDA/kg after three days of storage. This behavior indicates a pro-oxidant effect of alginate films, as the TBARS values were always higher than those of control samples without films. This result is quite impacting, as this effect of sodium alginate films in meat products was not presented so far. The chemical species involved in this pro-oxidant mechanism are difficult to assess due to the complex factors affecting oxidation reactions of meat lipids. Sodium chloride has been reported to increase the level of lipid oxidation, though the mechanism by which it occurs has not yet been clearly understood [62]. As the films of the present work were prepared using alginate sodium salt, sodium ions are present in the film’s matrix, along with the negatively charged polyelectrolyte nature of alginate molecules. This fact may have contributed for the pro-oxidant effect observed.

When the pineapple-peel antioxidants are added to alginate films in the form of encapsulated extract, peel juice microparticles or freeze dried peel powder, their antioxidant capacity effect seems to cancel the pro-oxidant effect of the alginate matrix itself, as lower TBARS values were observed for all samples. However, at 5 days of storage, there are no significant (*p* > 0.05) differences in TBARS values between AF and EF samples, and an increase in TBARS values was observed for samples with the other films (FDF and PJF), indicating that the inhibitory effect starts to disappear. Nevertheless, these results are quite positive since they show the ability of the pineapple peel antioxidants to delay meat lipid oxidation. It is envisaged a significant increase of their performance, if a higher amount is added in into the films, along with the use of a polymeric matrix presenting no pro-oxidant effect.

## 4. Conclusions

In this work, novel active films were developed and applied in the preservation of beef meat. It was possible to use pineapple peel as source of bioactive compounds in the development of edible alginate films with antioxidant and antimicrobial activities, produced exclusively from renewable and edible materials. The films containing encapsulated hydroalcoholic peel extracts presented a good performance in the inhibition of initial aerobic mesophilic bacteria growth in meat samples, on their advanced multiplication phase. Also, meat samples wrapped with the active films maintained their color in a good extent during five days under refrigeration (4 °C). The results suggest that the incorporation of pineapple peel antioxidants in alginate films has the potential to retard lipid oxidation in meat samples. However, attention must be paid to the observed pro-oxidant effect of the alginate matrix itself. The possibility of incorporation of a higher amount of pineapple peel bioactive compounds in the films, and of using a different biopolymer matrix, should be studied.

## Figures and Tables

**Table 1:** Antioxidant activity of alginate films with microparticles and pineapple peel powder.

Films	FRAP ^1^ μmol FeSO_4_.7H_2_O/g Dried Film
Alginate with extract microparticles (EF)	0.18 ± 0.02 ^b^
Alginate with peel juice microparticles (PJF)	0.15 ± 0.02 ^b^
Alginate with freeze-dried powder (FDF)	0.35 ± 0.04 ^a^
Alginate film (AF)	0.02 ± 0.01 ^c^

^1^ Results are the means of three determinations ± standard deviation. Different lowercase letters in the same column represent significantly different values determined by Tukey test (*p* < 0.05).
